# Combined Structural and Voltage Control of Giant Nonlinearities in Semiconductor Superlattices

**DOI:** 10.3390/nano11051287

**Published:** 2021-05-13

**Authors:** Mauro Fernandes Pereira, Apostolos Apostolakis

**Affiliations:** 1 Department of Physics, Khalifa University of Science and Technology, Abu Dhabi 127788, United Arab Emirates; 2 Institute of Physics, Czech Academy of Sciences, 18221 Prague, Czech Republic; apostolakis@fzu.cz

**Keywords:** gigahertz, terahertz, semiconductor superlattices, harmonic generation

## Abstract

Recent studies have predicted a strong increase in high harmonic emission in unbiased semiconductor superlattices due to asymmetric current flow. In parallel, an external static bias has led to orders of magnitude control of high harmonics. Here, we study how this control can affect the operation of superlattice multipliers in a range of input frequencies and powers delivered by commercially available GHz sources. We show that the strongly nonlinear behavior can lead to a very complex scenario. Furthermore, it is natural to ask what happens when we combine both asymmetry and voltage control effects. This question is answered by the simulations presented in this study. The efficiency of high-order even harmonics is increased by the combined effects. Furthermore, the development of ‘petals’ in high-order emission is shown to be more easily achieved, opening the possibility to very interesting fundamental physics studies and more efficient devices for the GHz–THz range.

## 1. Introduction

Semiconductor superlattices (SSLs) are nanomaterials constructed atom-by-atom by means of epitaxial growth techniques, and they make in many ways, the ideal system to study quantum transport and optics controlling both structural parameters and applied fields [1,2,3]. The experimental detection of coherent Bloch oscillations and Stark ladders [4] was the first step for the development of SSL multipliers (SSLMs) as sources and heterodyne detectors, generating higher order harmonics well within the far-infrared (54th at 37 μm) [5,6]. Compact sources operating at room temperature, such as superlattice electron devices (SLEDs), can deliver so far a 4.2 mW power output at an input frequency of 145 GHz [7]. These SLED structures can in principle be synchronized with the potential to become input sources with a much higher output power [8], which can be combined with SSLMs to create hand-held devices for a large number of state of the art gigahertz–terahertz spectroscopic techniques. This scenario can become even more interesting with quantum dot and graphene superlattices [9,10,11,12,13]. At present, quantum cascade lasers (QCLs) demonstrate high output power in the THz and mid-infrared (MIR) regions [14,15,16], which like the SSLMs are epitaxially grown and multilayered at nanoscale and can also be predictably simulated by a combination of Nonequilibrium Green’s functions, density matrix and Boltzmann equation methods [17,18,19,20,21,22,23,24,25,26,27,28]. QCL emission stems from intersubband optical transitions, in contrast with the mechanisms underlying high-order harmonic generation (HHG) in SSLMs, which we briefly summarize next. When an SSLM is biased, an input oscillating field can modulate the Bloch oscillations giving rise to HHG [29,30,31,32]. This nonlinear emission from the SSLM can be strongly affected by the involvement of electric field domains [33,34]. Strong excitonic effects can also play a role under well-defined conditions [35,36,37]. All these processed can be somehow be combined to enhance HHG in SSLMs; however, in this paper we focus on the combined effect of a uniform bias and current–voltage asymmetry on the Bloch oscillations modulation, which has successfully predicted giant voltage control of HHG in unbiased SSLMs [28,38,39,40,41,42].

## 2. Materials and Methods

The simulations presented in this paper are based on the optical response of a strongly coupled semiconductor superlattice excited by combined static and oscillating fields. Next we summarize a semiclassical approach, in which the electromagnetic field is treated classically, and the materials by quantum mechanics. The semiclassical approach can be adapted to other nonlinear voltage current cases; however, note that the equations used here are specific for superlattices. The derivation starts from Maxwell’s equations. Assuming linear polarization along $\hat{z}$, we do not need a vector notation. If there are no bound charges and no magnetization in the medium, we obtain the following wave equation:

(1) $$-\nabla^{2}E+\frac{1}{c^{2}}\frac{\partial^{2}E}{\partial t^{2}}=-\mu_{0}\frac{\partial J}{\partial t},$$

where the speed of light is $c^{2}=1/\mu_{0}\epsilon_{0}$. Simplifying the Boltzmann equation for carrier transport with relaxation approximation, one can show that if the superlattice is illuminated with combined static and monochromatic fields [1,28,38,39,40,41,42],

(2) $$E=E_{dc}+E_{ac}\cos\left(2\pi \nu t\right),$$

the nonlinear generation of harmonics and current rectification, with the current *I*, related to the current density by *j* = *I*/*A*, is described by the following:

(3) $$I=I_{dc}+\overset{\infty }{\underset{l=1}{\sum }}I_{l.c}\cos\left(2\pi l\nu t\right)+I_{l.s}\sin\left(2\pi lJt\right).\;I_{dc}=\overset{\infty }{\underset{𝓅=-\infty }{\sum }}J_{𝓅}^{2}\left(\alpha \right)Y\left(U\right)\;I_{l}^{c}=\overset{\infty }{\underset{𝓅=-\infty }{\sum }}J_{𝓅}\left(\alpha \right)\left[J_{𝓅+l}\left(a\right)+J_{𝓅-l}\left(a\right)\right]Y\left(U\right),\;I_{l}^{s}=\sum_{𝓅=-\infty }^{\infty }J_{𝓅}\left(\alpha \right)\left[J_{𝓅+l}\left(a\right)-J_{𝓅-l}\left(a\right)\right]K\left(U\right).$$

The strong nonlinear behavior is dictated mathematically by the Bessel functions of order 𝓅 and first kind, $J_{𝓅}$, ruled by $\alpha =eE_{ac}d/\left(h\nu \right)$, which controls the modulation of Bloch oscillations. In addition, $U=eE_{dc}d+𝓅h\nu$ denotes shifted potential drops by an integer number of photon quanta. The charge of an electron, superlattice period and Plank’s constant are given respectively by e,p,h.

The functions Y and K read as follows:

(4) $$Y\left(U\right)=j_{0}\frac{2U/\Gamma }{1+{\left(U/\Gamma \right)}^{2}},\;K\left(U\right)=\frac{2j_{0}}{1+{\left(U/\Gamma \right)}^{2}}.$$

The global dephasing is given by $\Gamma =\hbar /\tau =U_{c}$. It characterizes the region of negative differential resistance and $j_{0}$ denotes current maximum at $U=U_{c}=\Gamma$. Asymmetric flow in the voltage–current is described by an Ansatz solution introduced in Ref. [28],

(5) $$j_{0}=\{\begin{array}{c} j_{0}^{-},\;U<0\\ j_{0}^{+},\;U\geq 0 \end{array},\Gamma =\{\begin{array}{c} \Gamma^{-},\;U<0\\ \Gamma^{+},\;U\geq 0 \end{array}.$$

**Figure 1:**
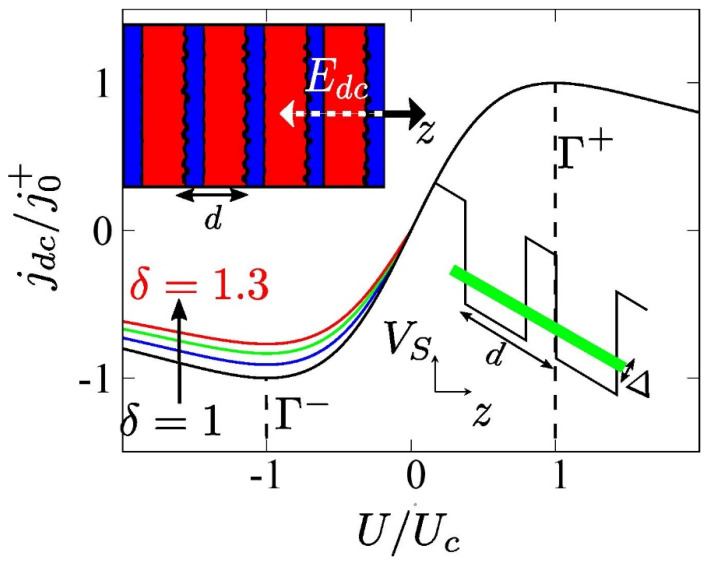
The current–voltage characteristics, $j_{dc}\left(U\right),$ in the absence of an oscillating field for different values of the asymmetry parameter, from bottom (black) to top (red) as follows: δ=1, 1.1, 1.2, 1.3. The left inset is a schematic representation of the semiconductor superlattice with a superlattice period, d, which results in the asymmetric current flow. The SSL is biased by a static electric field $E=\left(0,0,E_{dc}\right)$ antiparallel to the growth axis (−*z*) of the superlattice structure. The right inset corresponds to the tilted superlattice potential $V_{s}$ due to the application of the electric field. The shaded green region indicates the first miniband with width Δ.

Our calculations are performed for a strongly coupled superlattice consisting of alternating GaAs and AlAs semiconducting layers depicted by red and blue regions, respectively, in the left inset of Figure 1. Therefore, the tight-binding dispersion relation $\varepsilon \left(k_{z}\right)=-\frac{\Delta }{2}\cos k_{z}\;d$ can describe the kinetic energy of the electron in the first SSL miniband, where $k_{z}$ is the quasimomentum and Δ is the miniband width [1]. Note that typically the interfaces of GaAs over AlAs do not have the same quality as those of AlAs over GaAs. To characterize the quality of interfaces in the superlattice we employ the asymmetry parameter $\delta =\Gamma^{+}/\Gamma^{-}=j_{0}^{+}/j_{0}^{-}$, which effectively depends on the different interface roughness self-energies [39,41]. Therefore, as δ increases the peak of the current–voltage characteristic for positive bias practically remains the same, whereas for negative bias the peak is significantly reduced. The main input parameters of Equations (3)–(5) taken from Ref. [28] are as follows: $j_{0}^{+}=2.14\times 10^{9}\;A/m^{2}$, $\Gamma^{+}$ = 21 meV, resulting from Nonequilibrium Green’s functions (NEGF) calculations and L=121.4 nm corresponding to an SSL with 18 lattice periods (d) of 6.23 nm each, i.e., eighteen monolayers GaAs and four monolayers AlAs homogeneously doped with electron density $N=1.5\times 10^{18}\;{\mathrm{cm}}^{-3}$.

We assume next a slowly varying propagation, so that it is uniform throughout the active region (z-direction). Harmonic electromagnetic fields, current densities and polarizations will build up from zero and we can use a complex representation for plane waves inside a waveguide between *x* = 0 and *x* = *L*. In general,

(6) $$E=\sum_{l}E\left(x,l\omega \right)e^{-il\omega t},\;\vec{P}=\epsilon_{0}\left({𝓅}_{0}+\sum_{l}\chi_{1}\left(l\omega \right)E\left(x,l\omega \right)e^{-il\omega t}+\sum_{l}P_{NL}\left(x,l\omega \right)e^{-il\omega t}\right),\;\vec{j}=\sum_{l}j\left(x,l\omega \right)e^{-il\omega t}.$$

The equations connecting electric field, vector potential, current and the macroscopic polarization lead to the relations.

(7) $$\vec{E}=-\frac{\partial \vec{A}}{\partial t},\;\vec{j}=\frac{\partial \vec{P}}{\partial t}\Rightarrow E\left(x,l\omega \right)=\left(il\omega \right)A\left(x,l\omega \right),\;{𝒿}_{NL}\left(x,l\omega \right)=\epsilon_{0}\left(-il\omega \right)P_{NL}\left(x,l\omega \right)$$

where we subsequently insert Equation (7) into (wave) Equation (1).

(8) $$-\nabla^{2}\left(-\frac{\partial \vec{A}}{\partial t}\right)+\frac{1}{c^{2}}\frac{\partial^{2}}{\partial t^{2}}(-\frac{\partial \vec{A}}{\partial t})=-\mu_{0}\frac{\partial \vec{J}}{\partial t}=-\mu_{0}\frac{\partial^{2}\vec{P}}{\partial t^{2}}=-\mu_{0}\epsilon_{0}\frac{\partial^{2}\vec{P}}{\partial t^{2}}=-\frac{1}{c^{2}}\frac{\partial }{\partial t}\left(\frac{\partial^{2}\vec{P}}{\partial t}\right),$$

or equivalently,

(9) $$\nabla^{2}\vec{A}-\frac{1}{c^{2}}\frac{\partial^{2}\vec{A}}{\partial t^{2}}=-\frac{1}{c^{2}}\frac{\partial \vec{P}}{\partial t},$$

thus,

(10) $$\frac{\partial^{2}A(x,l\omega )}{\partial x^{2}}+\left(\frac{l\omega }{c}\right)A(x,l\omega )=\frac{\left(il\omega \right)}{c^{2}}[(il\omega )\chi (x,l\omega )A(x,l\omega )+P_{NL}(x,l\omega )].$$

Introducing the complex wavenumber $\kappa_{l\omega }$, $\kappa_{l\omega }^{2}={\left(\frac{l\omega }{c}\right)}^{2}\left(1+\chi_{1}\left(l\omega \right)\right)$,

(11) $$\frac{\partial^{2}A(x,l\omega )}{\partial x^{2}}+\kappa_{l\omega }{}^{2}A(x,l\omega )=\mu_{0}\epsilon_{0}(il\omega )P_{NL}(x,l\omega )=-\mu_{0}{𝒿}_{NL}(x,l\omega ).$$

At this point we introduce a propagation factor $A\left(x,l\omega \right)=e^{i\kappa_{l\omega }x}A\left(x,l\omega \right).$ The slowly varying amplitude approximation (SVEA), $\left|\kappa_{l\omega }A\right|\;\gg \;\left|\frac{\partial A}{\partial x}\right|$, simplifies the wave equation for the vector potential. Note that we are interested in l≥2 harmonics.

(12) $$2i\kappa_{l\omega }e^{i\kappa_{l\omega }x}\frac{\partial A\left(x,l\omega \right)}{\partial x}=-\mu_{0}{𝒿}_{NL}\left(x,l\omega \right).$$

The harmonic fields and vector potential build up from zero at *x* = 0, thus,

(13) $$A\left(L,l\omega \right)=-\mu_{0}\int_{0}^{L}\frac{1}{2i\kappa_{l\omega }}e^{-i\kappa_{l\omega }x}{𝒿}_{NL}\left(x,l\omega \right)dx.$$

In semiconductors, the real part of the linear wavenumber is typically much larger than its imaginary part. Thus (for $k_{\omega }\equiv k_{1\omega }$) we introduce the Ansatz ${𝒿}_{NL}\left(x,l\omega \right)={𝒿}_{l}e^{ilk_{\omega }x}$,

(14) $$A\left(L,l\omega \right)=-\mu_{0}{𝒿}_{l}\int_{0}^{L}\frac{1}{2i\kappa_{l\omega }}e^{i\kappa_{l\omega }\left(L-x\right)\;}e^{ilk_{\omega }x}\;dx=\mu_{0}{𝒿}_{l}\frac{e^{i\kappa_{l\omega }L}}{2\kappa_{l\omega }}\frac{1}{lk_{\omega }-\kappa_{l\omega }}\left[e^{iL\left(lk_{\omega }-\kappa_{l\omega }\right)}-1\right]$$

For $L\left(lk_{\omega }-\kappa_{l\omega }\right)\ll 1$, we have the following:

(15) $$A\left(L,l\omega \right)\approx i\mu_{0}{𝒿}_{l}\frac{L}{2\kappa_{l\omega }}e^{i\kappa_{l\omega }L}.$$

The real and imaginary components of $\kappa_{l\omega }$ are given by $ℛ\left\{\kappa_{l\omega }\right\}=n_{l\omega }\frac{l\omega }{c}$, $ℐ\left\{\kappa_{l\omega }\right\}=\kappa_{l\omega }^{\prime \prime }$, and typically $ℛ\left\{\kappa_{l\omega }\right\}\gg ℐ\left\{\kappa_{l\omega }\right\}$. Introducing the contact area S, the (averaged) power emitted by the $l^{th}$ order harmonic reads,

(16) $$P_{l}=\frac{n_{l\omega }\epsilon_{0}cS}{2}\langle {\left|E_{l\omega }\right|}^{2}\rangle =\frac{n_{l\omega }\epsilon_{0}cS}{2}{\left(l\omega \right)}^{2}\langle {\left|A\left(L,l\omega \right)\right|}^{2}\rangle =\frac{c\mu_{0}L^{2}S}{8n_{r}}\left[j_{l,c}^{2}+j_{l,s}^{2}\right],$$

where we have approximated the refractive index by the background refractive index $n_{r}$. Note that in our calculations we consider a contact area S∼ 50 μm^2^ corresponding to a typical SSL mesa structure with a diameter 8 μm.

## 3. Results

**Figure 2:**
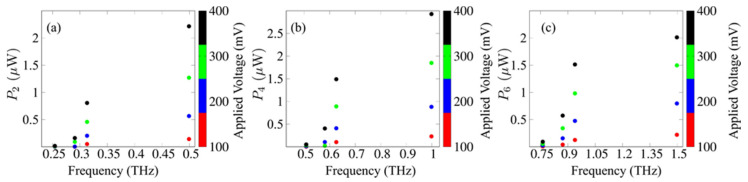
(**a**) Second, (**b**) fourth and (**c**) sixth harmonics output powers for SSL multipliers under the influence of a biased oscillating field for different input SLED devices over frequency ranges 250–1500 GHz for a perfectly symmetric current–voltage curve. The input frequencies and powers given respectively by 127.1, 145.3, 156.5, 249.6 GHz and 14, 4.2, 1.7, 0.92 mW.

**Figure 3:**
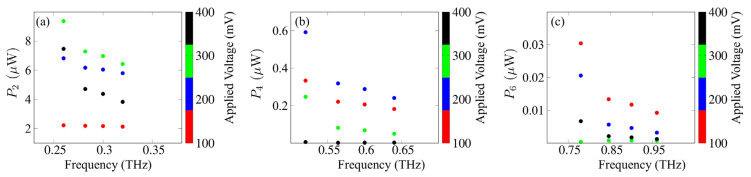
(**a**) Second, (**b**) fourth and (**c**) sixth harmonics output powers for SSL multipliers under the influence of a biased oscillating field for different input BWO devices over frequency ranges 250–950 GHz for a perfectly symmetric current–voltage curve. The input frequencies and powers given respectively by 130, 141, 150, 160 GHz and 61.6, 47, 45.2, 42.2 μW.

**Figure 4:**
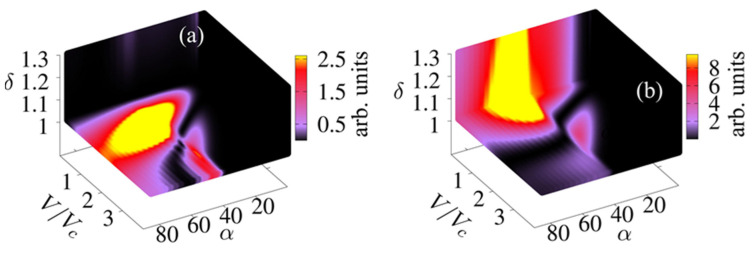
(**a**) Third harmonic (**a**) second and (**b**) third harmonics output powers (arb. units) in a superlattice with period d, as a function of $\alpha =\left(eE_{i}\right)d/\left(h\nu \right)$, *V* (voltage) and *δ* (asymmetry parameter). This parameter is directly proportional to the input electromagnetic field oscillating at *ν* = 249 GHz. The control voltage, $V/V_{c}$ is given as a ratio with respect to the critical voltage $V_{c}$, which characterizes negative differential resistance $U_{c}=eV_{c}$. The idea is to focus on the voltage, field and asymmetry control.

**Figure 5:**
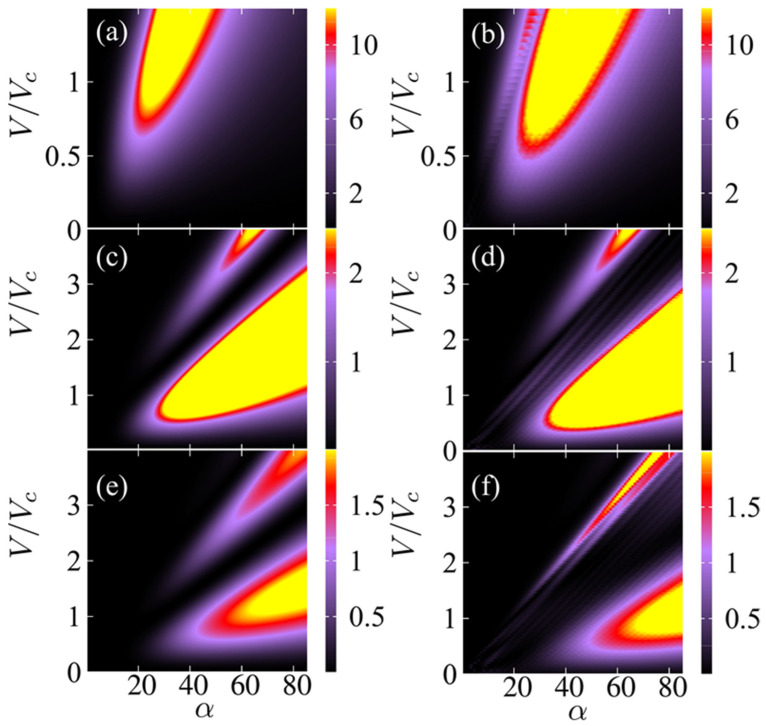
(**a**,**b**) Second, (**c**,**d**) fourth and (**e**,**f**) sixth harmonics output powers (arb. units) in a superlattice with period *d*, for *δ* = 1 (**a**,**c**,**e**) and *δ* = 1.3 (**b**,**d**,**f**), as a function of $\alpha =\left(eE_{i}\right)d/\left(h\nu \right)$, *V* (voltage) and *δ* (asymmetry parameter). The input electromagnetic field oscillates at *ν* = 249 GHz. The control voltage, $V/V_{c}$ is given as a ratio with respect to the critical voltage $V_{c}$, which characterizes negative differential resistance.

Figure 2 shows the output powers for the second, fourth and sixth harmonics of the SSLMs excited by well-defined SLED input powers and frequencies extracted from experiments [7] that were used for the comparison with different input device powers [4]. Figure 3 shows the corresponding analysis for backward wave oscillators (BWOs) [4,40]. Figure 3 shows the harmonics output power (arb. Units) in a superlattice with period d, as a function of $\alpha =\left(eE_{ac}d\right)/\left(h\nu \right)$, the applied voltage V and the asymmetry parameter $\delta =j_{0}^{+}/j_{0}^{-}$. The input parameters are connected by the relation $\frac{j_{0}^{+}}{\Gamma^{+}}=\frac{j_{0}^{-}}{\Gamma^{-}}$. The black areas designate the set of values (V, α, *δ*) for which $P_{l}$ demonstrates a weak or zero output (see Figure 4a,b). On the other hand, the colored areas reveal discrete ‘islands’ of enhanced harmonic response. Finally, in Figure 5 we fix the level of current asymmetry *δ* and look at details of the second, fourth and sixth harmonics. In this case, the colored areas indicate ‘petals’ of significant harmonic response as a function only of the voltage, V, and the parameter α, which is directly proportional to the amplitude of the oscillating field. The high harmonic ‘petals’ are separated by black areas which again are defined by the set of values (V, α) for which $P_{l}$ shows a small harmonic response.

## 4. Discussion

Figure 2 is based on specific frequencies and input powers delivered by available SLED sources [7,40]. It shows a monotonic increase in the output power with applied voltage. However, the scenario in Figure 3 for powers and frequencies typically delivered by BWOs [28,40] is far more complex. The maximum output for even harmonics occurs in a well-defined region of the input parameters, not necessarily at the largest applied voltage. This is consistent with the predictions and measurements in Ref. [42]. We also expect that current flow asymmetry improves the even harmonics, as predicted in Ref. [41]. Thus, the natural question to ask is, what happens when we combine both, controlling the output with both (V,δ). Figure 4 and Figure 5 answer this question. We see that the asymmetry allows for strong emission at a lower voltage. This may make the observation of high harmonic ‘petals’ easier, since the available samples, such as those used in the experiments described in Ref. [42], cannot sustain high voltages without permanent sample damage. This can also lead to devices with a more uniform higher power output at high-order even harmonic, leading to a wide range of useful frequencies in the GHz–THz range.

In conclusion, this paper describes the consequence of the combined intrinsic voltage flow asymmetry with an externally applied voltage, predicting an increase in efficiency for even harmonics and the possibility to more easily observe the development of ‘petals’ in high harmonic emission. This unusual nonlinear effect should open new possibilities for applications and fundamental physics studies.

## Data Availability

The data presented in this study are available on request from the corresponding author.
